# Role of Regulated Proteolysis in the Communication of Bacteria With the Environment

**DOI:** 10.3389/fmolb.2020.586497

**Published:** 2020-10-15

**Authors:** Sarah Wettstadt, María A. Llamas

**Affiliations:** Department of Environmental Protection, Estación Experimental del Zaidín-Consejo Superior de Investigaciones Científicas, Granada, Spain

**Keywords:** bacteria, proteolysis, signaling, gene regulation, transcription factor, sigma factor, two-component system

## Abstract

For bacteria to flourish in different niches, they need to sense signals from the environment and translate these into appropriate responses. Most bacterial signal transduction systems involve proteins that trigger the required response through the modification of gene transcription. These proteins are often produced in an inactive state that prevents their interaction with the RNA polymerase and/or the DNA in the absence of the inducing signal. Among other mechanisms, regulated proteolysis is becoming increasingly recognized as a key process in the modulation of the activity of these signal response proteins. Regulated proteolysis can either produce complete degradation or specific cleavage of the target protein, thus modifying its function. Because proteolysis is a fast process, the modulation of signaling proteins activity by this process allows for an immediate response to a given signal, which facilitates adaptation to the surrounding environment and bacterial survival. Moreover, regulated proteolysis is a fundamental process for the transmission of extracellular signals to the cytosol through the bacterial membranes. By a proteolytic mechanism known as regulated intramembrane proteolysis (RIP) transmembrane proteins are cleaved within the plane of the membrane to liberate a cytosolic domain or protein able to modify gene transcription. This allows the transmission of a signal present on one side of a membrane to the other side where the response is elicited. In this work, we review the role of regulated proteolysis in the bacterial communication with the environment through the modulation of the main bacterial signal transduction systems, namely one- and two-component systems, and alternative σ factors.

## Introduction

Proteolysis was long considered solely a mechanism of protein degradation to recycle amino acids in a slow and somewhat non-selective way. However, recent advances in the field have evidenced that in biological systems, proteolysis serves both as a cellular housekeeper (general proteolysis) and a modulator of regulatory pathways (regulated proteolysis) (Ehrmann and Clausen, 2004; Dougan, 2013). General proteolysis is important for the removal of misfolded or damaged proteins in a relatively non-specific manner, helping to preserve cell physiology. On the contrary, regulated proteolysis can produce the specific cleavage but also the complete degradation of selected proteins in response to intra- or extracellular signals. Regulated protein degradation is also referred to as processive proteolysis and allows a cell to get rid of a given protein, while protein cleavage is referred to as non-processive proteolysis or processing and produces a defined change in the activity of that protein (Jenal and Hengge-Aronis, 2003). Many proteins, both in eukaryotes and prokaryotes, including receptors, kinases, transcription factors, and structural components, are proteolytically altered to gain activity or to modify their functions (Brown et al., 2000; Gur et al., 2011; Konovalova et al., 2014). Proteolysis is a fast process able to alter the function of a protein instantaneously without the delay associated with activation or inhibition of transcription or translation mechanisms. Furthermore, proteolysis is able to eliminate a protein from a cell when it is no longer needed on a much faster time scale than for example the dilution of the protein as consequence of cell division would allow. As such a fast mechanism, regulation of protein activity by proteolysis is advantageous when a fast response is needed. This is especially the case during signal transduction that requires a quick response to a given signal in order for the cell to adapt and survive in the surrounding environment. In addition to mediating a fast response, regulated proteolysis is a sophisticated tool to overcome the biological problem of signal transfer between two cellular compartments. The field of cell signaling was expanded by the discovery that transmembrane proteins can be cleaved on either side or within the plane of the membrane to liberate a cytosolic domain or protein able to modulate gene transcription. This regulatory paradigm know as regulated intramembrane proteolysis (RIP) is commonly found in compartmental membranes in eukaryotes and the cytoplasmic membrane of prokaryotes, and allows the immediate transfer of a signal from an extracytosolic compartment to the cytosol (Brown et al., 2000). We review in this work the role of regulated proteolysis in the modulation of the main bacterial signal transduction systems, including one- and two-component systems, and alternative σ factors.

## Proteases Involved in Regulated Proteolysis in Bacteria

Proteolysis is typically achieved by proteases (or peptidases), a group of enzymes that hydrolyze peptide bonds and thus breakdown proteins or peptides. Proteases are classified according to their active site residue or ion that carries out catalysis, and they include serine, threonine, cysteine, glutamic, asparagine, aspartic, and metallo- proteases (a comprehensive classification of proteases is available through the MEROPS database^1^ Rawlings et al., 2014). Proteases do not attack their substrates at random but display high degrees of specificity that depend on a variety of factors, such as co-localization of the protease and the substrate, the regulation of protease activity by activation/inhibition processes, and the accessibility of the substrate cleavage site for the protease (Ehrmann and Clausen, 2004). Notably, specific cues often modulate these factors. For example, specific signals can modify protease activity by changing the structural properties of the active site either exposing or hiding it, or by activating adaptor proteins that feed the substrate to the protease. The accessibility of the protease to the cleavage site of the substrate can be also modified in response to specific signals that for example produces the unfolding of the substrate or the loss of an interaction partner.

General and regulated processive proteolysis of cytoplasmic proteins is often carried out by chaperone-protease complexes (reviewed in Kirstein et al., 2009; Gur et al., 2011). The chaperone belongs to the AAA + protein family and uses ATP hydrolysis to deliver the substrate to the protease. Examples of chaperones include ClpA, ClpX, ClpC, and HslU, which associate with ClpP and HslV serine and threonine proteases, respectively (Table 1). These complexes (e.g., ClpAP, ClpXP, ClpCP, HslUV) are known as ATP-dependent proteases. This group of proteases also includes the Lon and FtsH serine and metallo- proteases, respectively (Table 1), that contain the AAA + ATPase and the protease functions in the same polypeptide.

**TABLE 1:** Proteases discussed in this review.

**Protease families^a^**	**Subfamilies/examples**	**MEROPS family**
**Aspartyl proteases**		
Membrane-inserted endopeptidases	Presenilin	A22
Aspartyl endopeptidase	SpoIIGA	A36
**Metalloproteases**		
ATP-dependent metallo-endopeptidases	FtsH	M41
Intramembrane metallo-endopeptidases	RseP, SpoIVFB, RasP	M50
Membrane-bound bacterial endopeptidases	BlaR1, MecR1	M56
Metallo-endopeptidases	IrrE	M78
**Serine proteases**		
Serine endopeptidases	DegS, DegP	S1
Clp endopeptidases	ClpP	S14
ATP-dependent serine endopeptidases	Lon	S16
Signal peptidases	SipS, SipT, SipT	S26
C-terminal processing peptidases	Prc	S41
Membrane-bound serine endopeptidases	Rhomboid (e.g., GlpG)	S54
**Threonine proteases**		
Component peptidases of the proteasome	HslV	T1

*^*a*^According to the MEROPS database (https://www.ebi.ac.uk/merops/).*

The proteases involved in regulated non-processive proteolysis are more diverse and often substrate-specific. Increasingly recognized have been the intramembrane cleaving proteases (known as I-Clips) for their unique abilities to cleave peptide bonds within cellular membranes and their function in RIP of signaling pathways (reviewed in Urban and Freeman, 2002; Makinoshima and Glickman, 2006). Bacterial I-Clips are divided into aspartyl, serine, and zinc metallo- proteases. Aspartyl intramembrane proteases are exemplified by the eukaryotic presenilin (Table 1), which has been linked to Alzheimer’s disease. Serine intramembrane proteases include the rhomboid proteases, which contain six transmembrane domains and an active site cavity that opens to the periplasm, and are represented by *Escherichia coli* GlpG (Table 1; Wang et al., 2006). The zinc intramembrane metalloprotease family includes the so-called site-2 proteases (S2P) represented in bacteria by *E. coli* RseP and *Bacillus subtilis* SpoIVFB (Table 1) (reviewed in Kroos and Akiyama, 2013). S2P proteases share a conserved core domain containing at least three transmembrane domains, with the first and the third transmembrane segments containing the HExxH and LDG active site motifs, respectively (Kinch et al., 2006). Importantly, S2P proteases play a key role in RIP of several signaling pathways in bacteria, including one-component systems and those involving alternative σ factors (see below). These RIP cascades usually involve a site-1 protease that produces the substrate for the S2P protease. A site-1 cleavage is likely required because SP2 proteases, like RseP, contain PDZ domains that act as size-exclusion filters that only allow truncated, and thus smaller forms of the substrate, to enter the catalytic site of the protease (Hizukuri et al., 2014). Identified site-1 proteases involved in RIP of bacterial signaling pathways include the DegS and DegP serine endoproteases, SipS and SipT serine signal peptidases, and the Prc C-terminal processing serine protease (Table 1) (described below).

## Regulated Proteolysis in Modulating the Activity of One-Component Systems and Transcription Factors

One-component systems are the simplest structures for sensing cues and the predominant signaling mechanisms in bacteria (Ulrich et al., 2005). Canonical one-component systems are composed of a single cytoplasmic protein that carries out both signal recognition and response. These functions are performed by two different domains within the protein: the signal is recognized by the signal sensing or input domain, and the response is performed by the output domain, which often is a helix-turn-helix (HTH) domain able to bind to DNA thus activating or repressing gene transcription (Ulrich et al., 2005). Therefore, one-component proteins are also referred to as transcription factors or transcriptional regulators. According to their cytosolic location, one-component proteins mainly respond to signals produced in the cytosol or small molecules able to diffuse through the membrane. Signal input usually induces a conformational change that exposes the output domain allowing its binding to DNA. Activity of one-component proteins can be modulated by regulated proteolysis, which thus controls transcription of target genes in a fast but irreversible manner.

**FIGURE 1:**
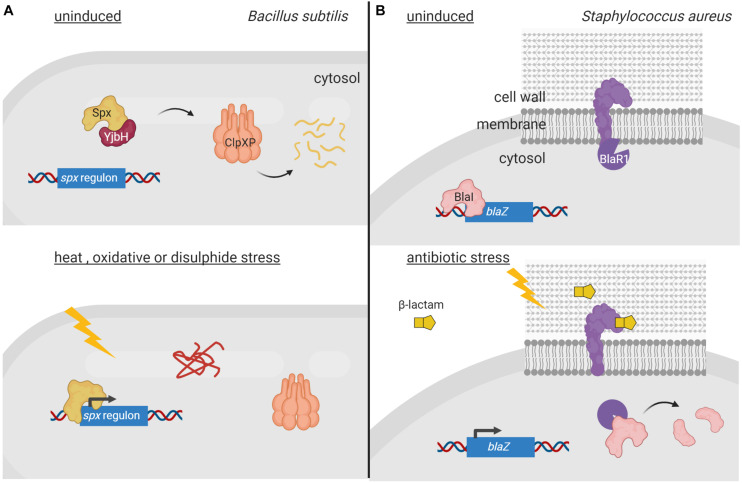
Regulated proteolysis of one-component proteins and transcription factors. **(A)** In non-stressed conditions, the transcription factor Spx (orange) is sequestered by YjbH (red) which delivers it to ClpXP (pink) for degradation. Upon heat, oxidative or disulfide stress, YjbH aggregates and releases Spx. Conformation change within ClpXP renders the protease unable to bind and digest Spx, resulting in transcription of the *spx* regulon. **(B)** In non-induced conditions, the membrane-bound protease BlaR1 (purple) is sequestered to the membrane while the repressor BlaI (orange) inhibits gene transcription. Extracellular presence of β-lactams triggers BlaR1 autocleavage and the release of its protease domain into the cytosol. BlaR1 cleaves BlaI thus terminating its inhibiting activity. This allows transcription of *blaZ*, among other genes, and thus production of β-lactamase that degrades the antibiotic. Figure produced with BioRender (BioRender.com).

Both regulated degradation and RIP of one-component proteins have been identified. Regulated degradation in response to specific signals usually occurs in the cytosol by the action of ATP-dependent proteolytic complexes (i.e., Clp). A well-studied example is the *Bacillus subtilis* Spx transcription factor, which controls thiol homeostasis, heat and oxidative response, and competence through both transcriptional activation and repression. Activity and concentration of Spx after exposure to stress is controlled by both the redox state of Spx and its ClpXP-mediated regulated proteolysis (Zuber, 2004; Figure 1A). The latter occurs through the activity of the YjbH adaptor protein, which in absence of stress binds to the C-terminus of Spx and delivers Spx to the ClpXP protease complex for degradation (Chan et al., 2014; Figure 1A). Since YjbH is highly prone to misfolding, under heat stress YjbH quickly aggregates rendering it unable to bind and deliver Spx to ClpXP (Engman and von Wachenfeldt, 2015). Moreover, under oxidative and disulfide stress, disulfide bond formation occurs in Spx, ClpXP and YjbH, leading to structural changes that renders Spx highly active, but inactivates ClpXP and promotes YjbH aggregation, thus avoiding Spx degradation (Rojas-Tapias and Helmann, 2018; Figure 1A). Similarly, the transcription factor FixK2 of the nitrogen fixing soybean endosymbiont *Bradyrhizobium japonicum* undergoes Clp-mediated regulated proteolysis in response to intracellular oxygen concentrations. Under microoxic conditions, FixK_2_ promotes transcription of genes required to adapt to low oxygen concentrations. In oxidative stress conditions, a cysteine near the DNA-binding domain of FixK_2_ is oxidized producing a conformational change that exposes the C-terminal 12 amino acids and renders FixK_2_ prone to ClpAP-mediated proteolysis (Bonnet et al., 2013).

Interestingly, some transcription factors are coupled with proteases that contain sensor domains and these two proteins form signal transduction pathways that modify gene transcription through regulated proteolysis. Such a signaling circuit is present in *Deinococcus* species, an extreme radiation-tolerant bacterium that employs the DrdO transcriptional repressor and the IrrE metalloprotease to respond to radiation and desiccation (Ludanyi et al., 2014). After exposure to radiation, signal transduction within IrrE occurs likely via its C-terminal GAF-like sensor domain resulting in activation of its N-terminal zinc-coordinating peptidase-like domain (Vujicic-Zagar et al., 2009). The peptidase domain then cleaves the DrdO repressor thus allowing transcription of *recA* and other DNA repair machine genes to adapt to radiation stress (Ludanyi et al., 2014).

Coupling can also occur between cytosolic transcription factors and membrane-bound proteases. In this case, the protease contains an extracytosolic signal sensing domain, which allows the one-component protein to respond to extracellular cues. As such, the activities of the transcriptional repressors BlaI of *Staphylococcus aureus* and MecI of methicillin-resistant *S. aureus* (MRSA) are controlled by the membrane-bound proteases BlaR1 and MecR1, respectively, in response to β-lactam antibiotics present on the outer surface of the bacterium (Zhang et al., 2001). BlaR1 and MecR1 are transmembrane proteins with a cytosolic N-terminal zinc metalloprotease domain and a C-terminal extracellular domain that resembles class-D β-lactamases and functions as a β-lactam sensor (Alexander et al., 2020). Presence of β-lactam antibiotics trigger acetylation of an active site serine located in the C-terminal sensor domains of BlaR1 and MecR1. Acetylation is transduced to the cytoplasmic domain leading to the activation of the metalloprotease domain and the degradation of the BlaI/MecI repressor. However, the mechanism behind this process is still unclear but it is likely similar for both repressors. It has been shown that acetylation of BlaR1 results in its autocleavage and release of the metalloprotease domain into the cytoplasm where it degrades BlaI (Llarrull et al., 2011; Figure 1B). A more recent model suggests that acetylation of MecR1 produces sterical overlap within the sensor domain and dislodgement of the last transmembrane helix that likely opens the cavity of the metalloprotease domain enabling substrate accessibility and catalysis (Belluzo et al., 2019). Nevertheless, degradation of the BlaI and MecI repressors by the proteases allows the transcription of (among others) the β-lactamase-encoding *blaZ* and the penicillin-binding protein PBP2a-encoding *mecA* genes, respectively, that confer resistance to β-lactam antibiotics (Llarrull et al., 2011; Belluzo et al., 2019; Figure 1B).

There are also membrane-bound one-component proteins that contain an extracytosolic signal-sensing domain and a cytosolic output domain, and regulated proteolysis is essential in activating these regulatory proteins. As such, the activities of the membrane-bound transcription activators ToxR and TcpP of the human pathogen *Vibrio cholerae* are controlled by both RIP and cytosolic regulated degradation. Under virulence−promoting conditions, e.g., presence of bile salts from the host, ToxR and TcpP, together with their respective interacting partners ToxS and TcpH, form a complex in the membrane that promotes the transcription of the *toxT* gene (Häse and Mekalanos, 1998). This gene encodes the ToxT transcriptional regulator, which is the direct activator of the two main virulence factors of *V. cholerae*, the cholera toxin and the toxin-co-regulated pilus (DiRita et al., 1991). Under conditions unfavorable for virulence gene activation, ToxR and TcpP undergo RIP. The RIP cascade of TcpP occurs in response to yet unknown signals that disrupt the TcpH/TcpP interaction. This exposes the periplasmic domain of TcpP to the C-terminal processing protease Prc that performs the site-1 cleavage of TcpP, which is followed by a site-2 transmembrane cleavage by the RseP protease (Matson and DiRita, 2005; Teoh et al., 2015). ToxR undergoes RIP in response to nutrient limitation at alkaline pH, which results in the reduction of two periplasmic cysteines of ToxR and the release of its interacting partner ToxS (Almagro-Moreno et al., 2015b; Lembke et al., 2018). This exposes ToxR to the action of the periplasmic site-1 serine proteases DegP and DegS, which produce the substrate for the site-2 protease RseP (Almagro-Moreno et al., 2015a,b). RIP of ToxR promotes the entrance of *V. cholerae* into a dormant environmentally persistent state (Almagro-Moreno et al., 2015a). Another membrane-bound one-component protein subjected to RIP is CadC of the pathogen *Salmonella enterica sv.* Typhimurium. CadC is an acid-sensing regulator that activates transcription of genes that contribute to the acid tolerance response of this pathogen (Lee et al., 2008). In response to low pH and lysine, the CadC periplasmic signal sensing domain is degraded by a yet unknown protease resulting in accumulation of the N-terminal DNA-binding domain in the cytoplasm where it can bind its target promoters. This leads to the activation of the acid stress response with production of the outer membrane porins OmpC and OmpF, among others (Lee et al., 2008).

## Modulation of Two-Component System Activity by Regulated Proteolysis

**FIGURE 2:**
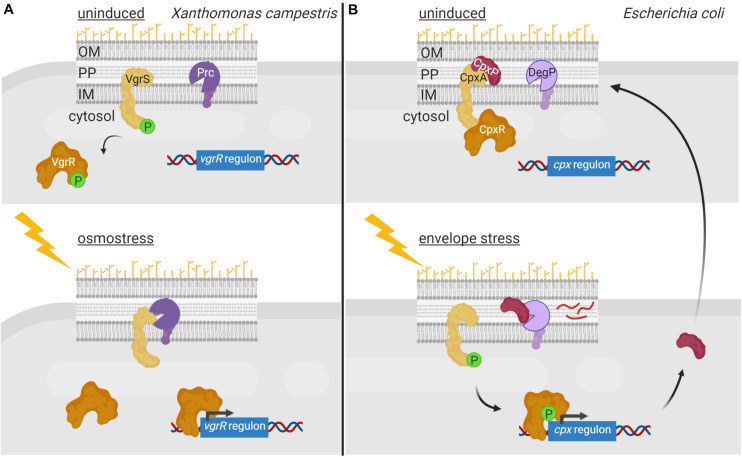
Regulated proteolysis of two-component systems. **(A)** In the uninduced state, the histidine kinase VgrS (yellow) is active and phosphorylates its cognate response regulator VgrR (orange) diminishing its affinity for the DNA. Upon osmostress, VgrS is cleaved by the periplasmic protease Prc (purple), which inhibits VgrS autokinase activity and VgrR phosphorylation. This increases the affinity of VgrR for its target promoters resulting in transcription of genes required to cope with osmostress. **(B)** In non-stressed condition, the histidine kinase CpxA (yellow) is bound to periplasmic regulator CpxP (red), which inhibits its kinase activity. Envelope stress triggers the degradation of CpxP by DegP (purple) and the activation of CpxA, which phosphorylates the response regulator CpxR (orange) triggering a transcription program to combat the envelope stress. Figure produced with BioRender (BioRender.com).

Two-component systems (TCSs) represent very efficient signal transduction pathways that allow bacteria to sense environmental changes and adapt to them in an appropriate manner. A canonical TCS is composed of a cytoplasmic membrane-bound histidine kinase that recognizes a specific stimulus, and a cytosolic response regulator that mediates the response (Stock et al., 2000). Activation of this signaling system in response to the inducing signal occurs via a phosphorelay cascade that leads to a His autophosphorylation of the histidine kinase and a subsequent Asp phosphorylation of the response regulator. Response regulators are DNA-binding proteins that modify gene transcription upon phosphorylation, thus translating the signal sensed by the histidine kinase into a response. Chemical ligands, environmental cues, phosphatases and auxiliary proteins directly adjust the phosphorylation levels of histidine kinases (Mascher et al., 2006). All these regulatory modes are reversible in controlling histidine kinase activity; however, irreversible proteolytic modification of histidine kinases also occurs. For example, in *Xanthomonas campestris*, proteolysis of the sensor histidine kinase VgrS is the signal required to modulate the DNA-binding activity of the cognate response regulator VgrR. In response to osmotic stress, the periplasmic PDZ-domain containing protease Prc directly binds to VgrS and cleaves its N-terminal periplasmic domain between residues Ala9 and Gln10 (Deng et al., 2018; Figure 2A). This terminates VgrS autokinase activity and halts VgrR phosphorylation. Unphosphorylated VgrR triggers transcription of stress-response genes required for the bacterium to resist osmostress. VgrR also promotes transcription of the *prc* gene, which results in a positive feedback loop within the regulatory cascade (Deng et al., 2018). The *X. campestris* VgrRS TCS is unusual for two reasons. First, the unphosphorylated form of VgrR binds to DNA with considerably higher affinity than the phosphorylated form (Deng et al., 2018), in contrast to most response regulators that are able to bind their target DNA only in their phosphorylated form (Stock et al., 2000). Second, while Prc is considered a C-terminal processing protease in *E. coli* and other Gram-negative bacteria (Chueh et al., 2019), it processes VgrS N-terminally (Deng et al., 2018). It is at present unclear how Prc is activated under osmostress in *X. campestris*. In *E. coli*, the PDZ domain of Prc stimulates protease activity by directly interacting with the inducing substrate (Chueh et al., 2019). However, in *X. campestris*, the Prc PDZ domain does not interact with the VgrS sensor domain (Deng et al., 2018). This difference could be the reason why Prc*^*Eco*^* recognizes and cleaves its substrates C-terminally, while Prc*^*Xca*^* cleaves within the N-terminal end of VgrS.

Regulated proteolysis also modulates activity of the *E. coli* CpxAR TCS that responds to cell envelope stress. The activity of this signaling system is controlled by the periplasmic regulator CpxP, which interacts with the CpxA histidine kinase preventing the activation of the CpxAR TCS pathway (Raivio and Silhavy, 1997; Figure 2B). In high-salt conditions, CpxP dissociates from CpxA while in conditions that lead to envelope stress or elevated pH, CpxP is degraded by the periplasmic protease DegP (Isaac et al., 2005; Tschauner et al., 2014; Figure 2B). CpxA liberation as well as changes in the lipid bilayer properties of the cytoplasmic membrane, activates CpxA autokinase function (Keller et al., 2015). This results in phosphorylation and thus activation of the CpxR response regulator (Isaac et al., 2005; Figure 2B). CpxR triggers the transcription of the *cpx* regulon, which includes gene products necessary to combat envelope stress (Isaac et al., 2005). Furthermore, the *cpxP* gene is part of this regulon and CpxP production allows shutting down the CpxAR pathway at the same time the cell is being relieved from the envelope stress. Similarly, proteolysis of the periplasmic regulator ExoR of the Gram-negative *Sinorhizobium meliloti* modulates activity of the ExoS/ChvI TCS, which controls the switch from free-living to invading cells. ExoR retains the membrane-bound sensor kinase ExoS in an off state by protecting it from degradation (Lu et al., 2012; Wiech et al., 2015). As soon as ExoR is cleaved within its N-terminus by yet unknown proteases, the ExoS histidine kinase domain is activated leading to ChvI phosphorylation (Lu et al., 2012; Wiech et al., 2015). This results in expression of ExoR as well as genes to shift toward a free-living lifestyle.

## Regulated Proteolysis in the Modulation of Alternative σ Factor Activity

Another important modulator of gene transcription in bacteria is the σ subunit of the RNA polymerase (RNAP). This dissociable subunit contains most promoter recognition determinants and directs the RNAP to the promoter region of the genes to be transcribed (Ishihama, 2000). Most bacteria contain a primary σ factor to transcribe general functions and a number of alternative σ factors to transcribe functions required only under specific conditions (Paget, 2015). Thus, the first step in the regulation of gene expression to shift toward a specific cell response often occurs through the substitution of the σ subunit of the RNAP (Ishihama, 2000). There are four different structural and functional groups of bacterial σ factors, with group I comprising primary σ factors and groups II to IV alternative σ factors (Paget, 2015). Group IV contains the so-called extracytoplasmic function σ factors (σ^ECF^), which is the largest and more diverse group, and is considered the third signaling mechanism in bacteria (Mascher, 2013; Sineva et al., 2017). Activity of alternative σ factors, especially that of σ^ECF^ factors, is often controlled through an inhibitory interaction with an anti-σ factor (Paget, 2015). Anti-σ factors keep the σ factor sequestered preventing its binding to the RNAP and only releasing it in response to a specific signal. There are cytosolic anti-σ factors that respond to intracellular signals although most anti-σ factors are single-pass transmembrane proteins that respond to extracytosolic signals. Importantly, regulated proteolysis plays a central role in the modulation of the activity of alternative σ factors by controlling the amount of σ factor in the cell or by allowing its release from the anti-σ factor in response to specific cues. The alternative σ factor, its cognate anti-σ factor or both can be subjected to regulated proteolysis.

### Regulated Proteolysis of Alternative σ Factors

**FIGURE 3:**
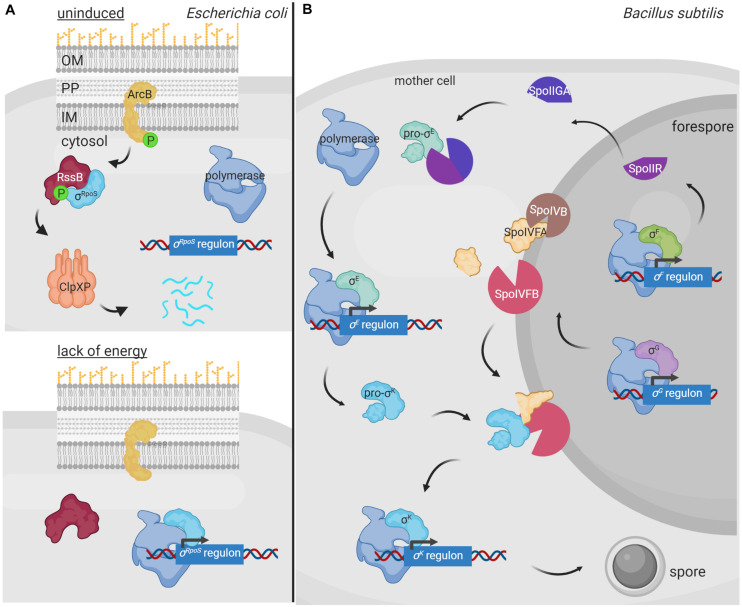
Regulated proteolysis in the modulation of alternative σ factor activity. **(A)** During *E. coli* exponential growth, the histidine kinase ArcB (yellow) phosphorylates RssB (red), which in turn binds to the σ^RpoS^ factor (cyan) and delivers it to the ClpXP proteolytic complex (pink) for degradation. Lack of energy during the stationary phase of growth blocks the kinase activity of ArcB and thus RssB phosphorylation. Unphosphorylated RssB cannot bind σ^RpoS^, and the σ factor is then able to bind the RNA polymerase (blue) and promotes transcription of the σ^RpoS^ regulon. **(B)** During spore formation in *B. subtilis*, a differential gene expression program is activated in the mother cell and the forespore by the action of the σ^SigF^, σ^SigE^, σ^SigG^ and σ^SigK^ factors. In the forespore, σ^SigF^ (σ^F^) promotes the transcription of SpoIIR (bright purple), which translocates into the mother cell and forms an active protease complex with SpoIIGA (dark purple) that produces the cleavage of the σ^SigE^ precursor pro-σ^SigE^ (pro-σ^E^) (green). Active σ^SigE^ initiates transcription of its regulon, including the σ^SigK^ precursor σ factor pro-σ^SigK^ (pro-σ^K^) (blue). Simultaneously, σ^SigG^ (σ^G^) promotes transcription of some of the proteases required to cleave pro-σ^SigK^, which includes the SpoIVB protease (brown) that cleaves the SpoIVFA protease (yellow) allowing it to form a complex with SpoIVFB (pink) able to cleave and activate σ^SigK^. Active σ^SigK^ triggers the final steps of spore formation. Figure produced with BioRender (BioRender.com).

Regulated proteolysis of alternative σ factors is for example used to prevent competition with the primary σ factor for binding to the RNAP during conditions in which the alternative σ factor is not necessary. This occurs in *E. coli* with the group II σ factor σ^RpoS^ that is the master regulator of the general stress response and essential for bacterial survival in the stationary phase as well as under a variety of stress conditions. σ^RpoS^ is always produced in the cell at basal levels but in exponentially growing bacteria it is hardly detectable due to proteolytic degradation by the ClpXP complex (Hengge, 2009). However, when cells enter the stationary phase, σ^RpoS^ accumulates and competes with the primary σ^70^ factor for binding to RNAP. σ^RpoS^ accumulation depends on different regulatory mechanisms acting at different levels, such as induction of *rpoS* gene transcription, increase of *rpoS* translation, and reduction of σ^RpoS^ proteolysis (Hengge, 2009). Besides the ClpXP proteolytic machine, σ^RpoS^ proteolysis requires the response regulator RssB that, when phosphorylated, functions as an anti-σ factor adaptor that interacts with σ^RpoS^ preventing its binding to the RNAP and delivering it to ClpXP for degradation (Stüdemann et al., 2003; Figure 3A). RssB is an orphan response regulator but it is phosphorylated by the ArcB histidine kinase in response to the energy state of the cell. When ArcB senses a lack of energy, its sensor kinase is inactivated (Malpica et al., 2004). This halts RssB phosphorylation decreasing its affinity for σ^RpoS^ and thus the proteolysis of this σ factor (Figure 3A). In these conditions, σ^RpoS^ can interact with the RNAP and promote transcription of the general stress response genes (Figure 3A).

In *B. subtilis*, proteolysis of alternative σ factors plays a key role during sporulation, a tightly regulated process that is activated when the nutrient availability is limited in the environment. Two different cellular compartments are formed during sporulation, the forespore and the mother cell, and differential gene expression in these compartments is governed by the successive appearance of four group III σ factors, namely σ^SigF^ and σ^SigG^ in the forespore, and σ^SigE^ and σ^SigK^ in the mother cell (Fimlaid and Shen, 2015). σ^SigE^ and σ^SigK^ are produced as inactive precursor peptides pro-σ^SigE^ and pro-σ^SigK^ factors and are activated by regulated proteolysis at specific points during sporulation (Figure 3B). Sporulation begins with the activation of the transcription factor Spo0A by phosphorylation through a phosphorelay cascade involving several histidine kinases sensing nutrient starvation conditions (Burbulys et al., 1991; Jiang et al., 2000). Phosphorylated Spo0A promotes transcription of about 120 sporulation genes, including those encoding the pro-σ^SigE^ and σ^SigF^ factors (Molle et al., 2003). The latter is synthesized together with a sequestering anti-σ factor SpoIIAB and gets activated after polar septum formation (Duncan et al., 1995). Active σ^SigF^ triggers the transcription of a DNA translocase complex that pumps the chromosome into the forespore, and that of the *sigG* σ factor and *spoIIR* genes (Fujita and Losick, 2002). SpoIIR is secreted into the intermembrane space and activates the aspartic protease SpoIIGA, which subsequently cleaves pro-σ^SigE^ activating this σ factor (Imamura et al., 2008). In the mother cell, σ^SigE^ promotes transcription of the gene encoding the membrane-associated pro-σ^SigK^ factor and that of a hydrolase complex required for the assembly of a channel or “feeding tube” between the mother cell and the forespore that is also necessary for σ^SigG^ activity (Camp and Losick, 2009; Morlot et al., 2010). Activated σ^SigG^ promotes transcription of determinants needed in the proteolytic cascade that leads to the σ^SigK^ activation. In this way, σ^SigK^ activation in the mother cell is coupled to σ^SigG^ activity in the forespore. Cleavage and activation of pro-σ^SigK^ requires the S2P intermembrane metalloprotease SpoIVFB, which gets itself activated through the proteolysis of its inhibitory factor SpoIVFA (Campo and Rudner, 2006; Figure 3B). Active σ^SigK^ triggers a gene expression program that results in complete sporulation formation (Ramirez-Guadiana et al., 2018).

### Regulated Proteolysis of Anti-σ Factors

Regulated proteolysis of an anti-σ factor in response to a signal is often used to liberate and activate the alternative σ factor, especially in the case of σ^ECF^ associated anti-σ factors. Cytosolic anti-σ factors are often processed by ATP-dependent proteases (i.e., Clp, Lon) (Table 1). For instance, the *Streptomyces coelicolor* σ^SigT^ factor is sequestered and protected by its cytosolic anti-σ factor RstA and both undergo regulated proteolysis by cytosolic proteases at different time points of cell growth (Mao et al., 2013). Regulated proteolysis of RstA by yet unknown proteases during the onset of secondary metabolism liberates σ^SigT^, which leads to a ClpP1/P2-dependent degradation of this σ factor (Mao et al., 2013). Because σ^SigT^ negatively regulates cell differentiation and *clpP1P2* expression, degradation of this σ factor results in production of secondary metabolites and the ClpP1/P2 proteolytic complex, which further accelerate σ^SigT^ degradation (Mao et al., 2013). The two-step proteolysis of σ^SigT^ by degrading first its cognate anti-σ factor RstA likely allows σ^SigT^ liberation when its function is required while subsequent σ^SigT^ degradation shuts off gene expression as soon as those conditions passed.

**FIGURE 4:**
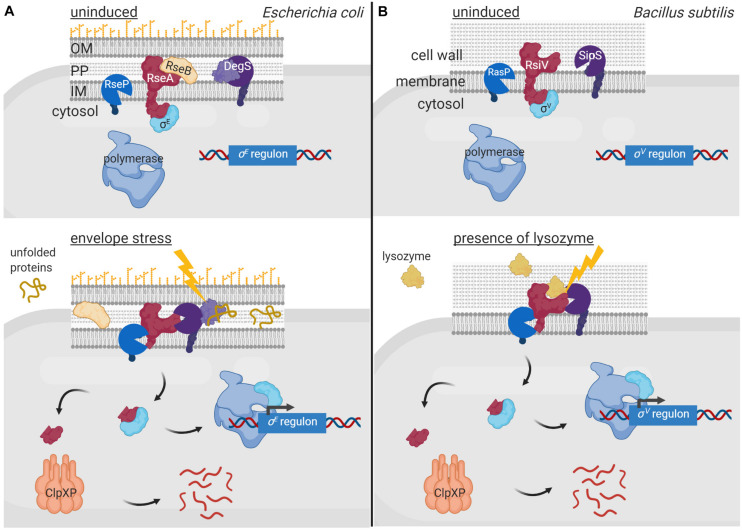
Regulated proteolysis in the activation of σ^ECF^ factors. In uninduced conditions, the membrane bound anti-σ factors RseA of *E. coli* **(A)** and RsiV of *B. subtilis* **(B)** (red) sequester and keep inactive their cognate σ^ECF^ factors σ^E^ **(A)** and σ^V^ **(B)** (cyan). Sequestration of σ^E^ by RseA is enhanced by RseB (yellow), which binds to and impedes RseA proteolysis. Activation of the σ^E^ and σ^V^ factors occurs in response to envelope stress and presence of lysozyme, respectively, by RIP and cytosolic proteolysis of the anti-σ factors. The DegS protease **(A)** and the SipS peptidase **(B)** (purple) perform the site-1 cleavage of the RIP cascade while the RseP **(A)** and RasP **(B)** proteases (blue) the site-2 cleavage. The cytosolic proteolysis is carried out by the ClpXP complex (pink). The signal activating the proteolytic cascade of RseA is unfolded periplasmic peptides and is sensed by the DegS site-1 protease **(A)**, while that of RsiV is lysozyme and is sensed by the anti-σ factor itself **(B)**. Figure produced with BioRender (BioRender.com).

Membrane-bound anti-σ factors are usually processed by RIP in response to an inducing signal (Heinrich and Wiegert, 2009). Indeed, the periplasmic site-1 protease of a RIP pathway sometimes even functions as the sensor protein that triggers the activation of the σ^ECF^/anti-σ factor signaling cascade. Generally, such protease contains one or multiple PDZ domains that occlude the catalytic site in absence of the signal and undergo conformational changes upon signal sensing exposing the catalytic site. The first and best characterized examples of such signaling pathway are the pathways activating the RpoE-like σ^ECF^ factors σ^E^ of *E. coli* and σ^AlgU^ of *P. aeruginosa* (reviewed in Ades, 2008; Chevalier et al., 2019; Otero-Asman et al., 2019b; Figure 4A). In absence of cell envelope stress, these σ factors are sequestered by their cognate membrane-bound anti-σ factors RseA and MucA. These inhibitions are enhanced by the periplasmic regulators RseB and MucB that bind to the periplasmic domains of RseA and MucA, respectively, protecting them from proteolysis. Activation of the σ^E^/RseA and σ^AlgU^/MucA signaling pathways occurs in response to cell envelope stress that leads to the accumulation of misfolded outer membrane proteins. Unfolded periplasmic peptides bind to the PDZ domain of the site-1 protease DegS (also known as AlgW in *P. aeruginosa*), being thus the signal that initiates the RIP pathway (Figure 4A). Signal binding reorientates the PDZ domain of DegS exposing its catalytic site (Walsh et al., 2003; Wilken et al., 2004). Simultaneously, LPS accumulation in the periplasm displaces the regulators RseB and MucB from RseA and MucA, respectively (Lima et al., 2013), allowing the binding of DegS to the anti-σ factors and their subsequent site-1 cleavage (Figure 4A). This cleavage generates the substrate for the site-2 protease of the RIP pathway, the metalloprotease RseP (also named MucP in *P. aeruginosa*) (Figure 4A). RseP cleaves within the transmembrane domain of the RseA/MucA anti-σ factors releasing their cytosolic N-domains bound to the σ^E^/σ^AlgU^ factor into the cytosol (Figure 4A). The RseA/MucA N-domain is subsequently degraded by the ClpXP protease, which completely liberate σ^E^ and σ^AlgU^ allowing their interaction with the RNAP and the transcription of the σ^E^/σ^AlgU^ regulon genes (Flynn et al., 2004; Qiu et al., 2008; Figure 4A). Importantly, the site-1 protease DegS does not only act as the sensor protein in these signaling pathways, but its activity is also the rate-limiting step in the regulatory cascade controlling σ^E^ and σ^AlgU^ activity (Chaba et al., 2007). Active σ^E^ and σ^AlgU^ factors promote transcription of genes to adapt to and protect the bacteria against periplasmic stress (Rhodius et al., 2006; Wood and Ohman, 2012). Besides, σ^AlgU^ is recognized for promoting the production of the exopolysaccharide alginate, which is responsible for the clinically relevant mucoid phenotype of *P. aeruginosa* that contributes to the persistence of this human pathogen in chronic infections (Ramsey and Wozniak, 2005).

In other membrane-associated σ^ECF^/anti-σ signaling systems, the anti-σ factor is the sensor protein and undergoes a conformational change upon signal recognition that allows the site-1 cleavage. The best characterized example of such signal transduction network is the σ^SigV^/RsiV system of *Bacillus subtilis* (reviewed in Ho and Ellermeier, 2019; Figure 4B). The membrane-bound protein RsiV does not only function as an anti-σ factor but also as a receptor for lysozyme (Hastie et al., 2016), which is an important component of the innate immune system of many organisms (Ragland and Criss, 2017). Binding of lysozyme to RsiV triggers RsiV degradation through a RIP cascade that leads to the activation of σ^SigV^ and the transcription of genes that confer resistance to lysozyme (Figure 4B). Importantly, the site-1 cleavage of RsiV is not carried out by a specific protease but by signal peptidases, especially SipS and SipT (Castro et al., 2018), which are the major signal peptidases of *B. subtilis*. The activity of signal peptidases is not regulated but constitutive, and RsiV cleavage in absence of lysozyme is prevented by two amphipathic helices that occlude the cleavage site (Lewerke et al., 2018). It has been hypothesized that binding of lysozyme to RsiV pulls the amphipathic helix into a β−sheet conformation, which exposes the cleavage site for the signal peptidases (Ho and Ellermeier, 2019). The site-2 cleavage of RsiV is performed by RasP, a membrane-embedded metalloprotease homologous to *E. coli* RseP (Figures 4A,B). RasP cleaves within the transmembrane domain of RsiV releasing the RsiV N-terminal cytosolic domain bound to the σ^SigV^ factor into the cytosol. Similarly to RseA and MucA (Figure 4A), it is assumed that the N-domain of RsiV is degraded by cytosolic proteases although they have not been identified yet (Ho and Ellermeier, 2019).

**FIGURE 5:**
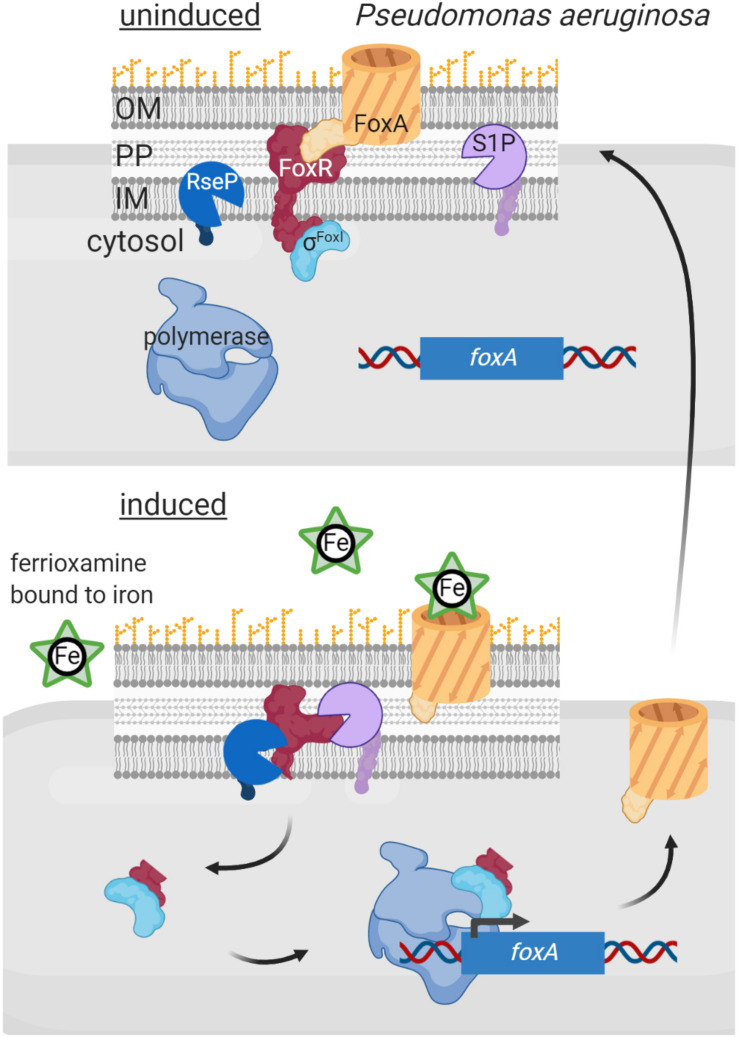
Regulated intramembrane proteolysis of CSS anti-σ factors. In uninduced conditions, the CSS anti-σ factor FoxR (red) keeps σ^FoxI^ (cyan) sequestered and inactive. In this situation RIP of FoxR is likely prevented through the interaction with the CSS outer membrane receptor FoxA (orange). Binding of the siderophore ferrioxamine to FoxA likely modifies the FoxR/FoxA interactions surface, which exposes the anti-σ factor to the action of the proteases of the RIP cascade. FoxR is cleaved by a still unknown site-1 protease (purple) and by the site-2 protease RseP (blue), so that the N-terminal domain of FoxR is released into the cytosol bound to σ^FoxI^. This complex interacts with the RNA polymerase (blue) and triggers transcription of the σ^FoxI^ regulon, including the *foxA* gene to increase the amount of the receptor in the outer membrane. Figure produced with BioRender (BioRender.com).

An additional layer of complexity in signal sensing and proteolytic activation of σ^ECF^/anti-σ factor systems occurs in Gram-negative bacteria, as *Pseudomonas*, in which several σ^ECF^/anti-σ proteins are functionally associated with an outer membrane receptor. These three proteins form a signal transduction system known as cell-surface signaling (CSS) that is activated by regulated proteolysis in response to a signal (reviewed in Llamas et al., 2014; Otero-Asman et al., 2019b; Figure 5). In these pathways, the outer membrane receptor, which belongs to the TonB-dependent transporter (TBDT) family, is the sensor protein and thus these systems are able to sense and respond to extracellular signals. CSS receptors function both as transporters of the inducing signal (often an iron-chelating compound, e.g., a siderophore) and as signal transfer proteins. This last function resides in the periplasmic N-terminal signaling domain (SD) of the CSS receptor that interacts with the periplasmic domain of its cognate membrane-bound anti-σ factor. In the current CSS model, signal binding at the outside of the receptor triggers a conformational change that modifies the SD/anti-σ factor interaction. This likely exposes the cleavage site of the anti-σ factor allowing its site-1 cleavage by a yet unknown periplasmic protease (Figure 5). The C-terminal processing periplasmic protease Prc seems to mediate the site-1 cleavage of a unique CSS protein of *P. putida*, named IutY, in which the σ^ECF^ and anti-σ factor functions are fused in a single protein (Bastiaansen et al., 2014; Bastiaansen et al., 2017). However, although required for CSS activation (Llamas et al., 2014; Otero-Asman et al., 2019a), whether Prc is directly or indirectly involved in cleaving CSS anti-σ factor proteins that are not fused to σ^ECF^ proteins is still unknown. Nevertheless, it is clear that a site-1 cleavage is necessary to generate the substrate for the site-2 protease, which for CSS anti-σ factors is the metalloprotease RseP (Draper et al., 2011; Bastiaansen et al., 2014; Figure 5). RseP cleavage leads to the release of the N-terminal cytosolic domain of the CSS anti-σ factor bound to its cognate σ^ECF^ factor into the cytosol (Figure 5). In some CSS pathways, this anti-σ factor domain is proteolytically removed by cytoplasmic proteases (e.g., ClpP). The best studied example of a CSS anti-σ factor subjected to cytosolic degradation is FpvR of *P. aeruginosa.* In response to the binding of the siderophore pyoverdine to the CSS receptor FpvA, FpvR undergoes regulated proteolysis liberating the σ^FpvI^ and σ^PvdS^ factors into the cytosol (Beare et al., 2003). RIP of FpvR is carried out by an unknown site-1 protease and by the site-2 protease RseP, and its cytosolic proteolysis by the ClpP protease (Draper et al., 2011; Bishop et al., 2017). However, in several pathways the N-terminal cytosolic domain of the anti-σ factor is not degraded and is required for CSS σ^ECF^ activity, having thus pro-σ activity (Figure 5). The best studied examples of CSS anti-σ factors with pro-σ activity include FoxR, FiuR and HxuR, which undergo RIP in response to the binding of the iron-chelating compounds ferrioxamine, ferrichrome and heme, respectively, to the CSS receptors FoxA, FiuA and HxuA, respectively (Llamas et al., 2006; Mettrick and Lamont, 2009; Bastiaansen et al., 2015; Otero-Asman et al., 2019a). Cleavage of these anti-σ factors by an unknown site-1 and the site-2 protease RseP releases their N-terminal cytosolic domains bound to their cognate σ^ECF^ factors, σ^FoxI^, σ^FiuI^ and σ^HxuI^, respectively (Figure 5). This anti-σ factor domain is thought to be bound to the σ^ECF^-RNAP holoenzyme during the transcription process (Llamas et al., 2014; Otero-Asman et al., 2019b; Figure 5).

## Concluding Remarks and Prospects

To communicate with the surrounding environment, bacteria contain tightly regulated and complex signaling pathways that allow them to perceive changes and perform adequate responses. While modulation of the activity of signal transduction systems by post-translational modifications like phosphorylation or methylation has been long known, the involvement of proteolysis in this process has become evident more recently. In fact, the idea of proteolysis as a regulatory mechanism has long been rejected because of the waste associated with destroying a protein synthesized at an energy cost. However, it has become evident that energy costs are less of a concern for a cell in the context of the regulation of its metabolic and signaling pathways. A major advantage of proteolysis as a regulatory post-transcriptional modification is that proteolysis is irreversible, which allows for an immediate response. It is therefore not surprising that the activity of many signal transduction proteins, which need to respond quickly to a stimulus (e.g., sudden envelope stress), is controlled by proteolysis. Moreover, the fact that it is an irreversible process allows for a longer duration of a response, which may be beneficial in developmental processes (e.g., sporulation). As outlined here, the signaling protein subject to regulated proteolysis can be the signal response protein directly or a signal transfer protein. Also, dependent on the signal transduction system, the activity of the regulatory protease can have stimulating or inhibiting effects on downstream gene transcription. Proteolysis of signal response proteins occurs either in the absence of the inducing stimulus to prevent the activity of a transcriptional activator, as exemplified by σ^RpoS^ (Figure 3A), or in response to the signal to inhibit the activity of a transcriptional repressor e.g., BlaI (Figure 1B) or to activate an inactive precursor protein as occurs with pro-σ^SigE^ and pro-σ^SigK^ (Figure 3B). Proteolysis of signal transfer proteins or that of interaction partners of these proteins usually occurs in response to the signal leading to the activation of a transcriptional activator. This is exemplified by the RIP of anti-σ factors that produces the activation of σ^ECF^ factors (Figures 4, 5) or the proteolysis of histidine kinase interaction partners leading to the activation of the cognate response regulator (Figure 2).

An important aspect of the regulation of signaling systems by proteolysis is the involvement of regulatory proteases in signal sensing, as described for BlaR1 (Figure 1B) and DegS (Figure 4A). BlaR1 is actually a fusion protein with a penicillin-binding and a zinc metalloprotease domain. The BlaR1 sensor domain resembles class-D β-lactamases and contains the three catalytic motifs found in all penicillin binding proteins (Alexander et al., 2020). A key difference between the BlaR1/MecR1 sensor domains and β-lactamases is their deacetylation rates, which is considerably slower in the proteases and likely necessary for proper signal transmission (Alexander et al., 2020). In contrast, the sensor domain of DegS serine protease is the PDZ domain located in the periplasmic domain of the protease. PDZ is a modular domain of about 80–100 amino acids found in proteins of all organisms that often recognize short amino acid motifs at the C-termini of target proteins (Lee and Zheng, 2010). C-terminal peptides of unassembled outer membrane proteins are known to bind to the PDZ domain of DegS, initiating a steric clash between the PDZ domain and the L3 loop of DegS that breaks the L3-mediated autoinhibition of the proteolytic active site (De Regt et al., 2015). This allows the allosteric activation of DegS by the binding of peptides. Other proteases involved in signaling also contain PDZ domains, e.g., C-terminal processing proteases (Chueh et al., 2019). It would be interesting to determine whether this domain is involved in signal sensing in these proteases and their mechanism of action.

In all, regulated proteolysis is a powerful tool to allow a bacterium to respond to environmental signals facilitating these simple life forms to thrive in highly diverse environments. Importantly, inhibition of signal transduction mechanisms is an interesting strategy for drug development that would prevent pathogens to detect and respond to the host environment. Because proteases are known to be druggable proteins, regulatory proteases involved in modulation of the activity of signal transduction systems required for pathogen’s survival represent excellent drug targets. In fact, an inhibitor of the site-2 RseP protease has already shown to considerably decrease *E. coli* survival (Konovalova et al., 2018). The identification of new regulatory proteases involved in critical bacterial processes thus holds promise for the development of novel antibacterials.

## Author Contributions

SW and ML wrote the manuscript. All authors contributed to the article and approved the submitted version.

## Conflict of Interest

The authors declare that the research was conducted in the absence of any commercial or financial relationships that could be construed as a potential conflict of interest.

## Footnotes

1 https://www.ebi.ac.uk/merops/
